# Evaluating integrated models of care

**DOI:** 10.1080/17571472.2017.1317406

**Published:** 2017-04-25

**Authors:** Paul Thomas

This Issue of LJPC (9.3) follows hot on the heels of the very successful (London) City Health Conference at which 160 participants and primary care leaders debated how to practically achieve New Models of Care that integrate care and health promotion at local, community level.

This Issue contains two papers that show different approaches to evaluation of integrated working:

• Morton et al. examined the records of their practice patients who attended Emergency Departments in a twelve month period. Only 25% saw their GP before attending and 95% of these needed specialist interventions. On the other hand 75% of patients self-referred and only 33% of these required specialist intervention.
• Cox et al. provide the first ever analysis of Specialist Palliative Care (SPC) services in London. All 50 SPC services provided data. This revealed that SPC service across London is still below the minimum service level set by NICE in 2004, including out of hours service. Also, SPC services see mostly cancer patients and there may be unmet need for SPC amongst the non-cancer population.

Also in this Issue:

• Avari and Devendra present a case study of a patient with HIV and diabetes. The discussion beautifully reveals the complexity of managing such a patient. Diabetes is four times more common in HIV-infected men exposed to anti retroviral therapy than in HIV seronegative men. Protease inhibitors based antiretroviral treatment is most commonly associated with increased incidence of diabetes. HbA1c is not recommended for screening for diabetes in HIV infected individuals. Fasting serum glucose and lipid levels should be measured in HIV-positive patients before beginning antiretroviral therapy.
• Carelli provides us with another fascinating view of medicine through the eyes of artists, this time a painting of a ‘Yawning Man’ by the Flemish painter Pieter Brueghel the Elder.

LJPC welcomes papers that help to understand how to evaluate integrated care. Keep your papers coming.

Paul Thomas Paul.thomas7@nhs.net

